# Morphological analysis of descending tracts in mouse spinal cord using tissue clearing, tissue expansion and tiling light sheet microscopy techniques

**DOI:** 10.1038/s41598-023-43610-z

**Published:** 2023-09-30

**Authors:** Jiongfang Xie, Ruili Feng, Yanlu Chen, Liang Gao

**Affiliations:** 1 Fudan University, Shanghai, 200433 China; 2 Key Laboratory of Structural Biology of Zhejiang Province, School of Life Sciences, Westlake University, Hangzhou, 310024 Zhejiang China; 3 Westlake Laboratory of Life Sciences and Biomedicine, Hangzhou, 310024 China

**Keywords:** Biological techniques, Neuroscience

## Abstract

Descending tracts carry motor signals from the brain to spinal cord. However, few previous studies show the full view of the long tracts from a 3D perspective. In this study, we have followed five less well-known tracts that project from midbrain, hindbrain, and cerebellum to the mouse spinal cord, using the tissue clearing method in combination with tiling light sheet microscopy. By tracing axons in spinal cord, we found several notable features: among the five tracts the collateral "sister" branches occurred only in the axons originating from the cerebellospinal tracts; the axons from the spinal trigeminal nucleus crossed the midline of spinal cord to the contralateral side; those arising in the medullary reticular formation ventral part gave many branches in both cervical and lumbar segments; the axons from superior colliculus terminated only at upper cervical but with abundant branches in the hindbrain. Furthermore, we investigated the monosynaptic connections between the tracts and motor neurons in the spinal cord through hydrogel-based tissue expansion, and found several monosynaptic connections between the medullary reticular formation ventral part axons and spinal motor neurons. We believe that this is the first study to show the full 3D scope of the projection patterns and axonal morphologies of these five descending tracts to the mouse spinal cord. In addition, we have developed a new method for future study of descending tracts by three-dimensional imaging.

## Introduction

Movement initiation and accomplishment depend on the function of descending tracts that act directly on the spinal cord, which originated from various brain areas, including the cerebral cortex^1,2^, midbrain^3^, hindbrain^4^ and cerebellum^5–7^. These pathways in mammals play vital roles in the initiation of movements of the limbs and trunk, including grasping, walking and posture maintenance. More than twenty brain regions have been identified giving rise to the descending tracts by using retrograde tracing methods (e.g., adeno-associated virus or dyes) in previous research^8,9^, in which the descending tracts arising from the cerebral cortex^1,2,10,11^, red nucleus^12,13^, hindbrain reticular formation^14,15^, and vestibular nuclei ^16^ have been studied extensively. However, several other descending nuclei, such as the medial and interposed cerebellar nuclei (Med and Int)^5,17^, the superior colliculus^18,19^, and the spinal trigeminal nucleus^20,21^ are less well understood.

Most current knowledges regarding the projection morphology of the descending tracts were obtained by 2D optical imaging of discrete tissue slices along the spinal cord. Obviously, 2D images of discrete transverse tissue slices can hardly reflect the 3D morphological characteristics of the tracts besides the time consuming and laborious sample preparation and imaging processes. Furthermore, the obtained descending tract images are insufficient to study the connectome between the descending neurons and the spinal cord neurons due to opaqueness of tissue slices and the limited spatial resolution of the imaging techniques at the time.

The development of tissue clearing^22,23^ and light sheet microscopy techniques^19^ offers a new solution to solve the above problems. In general, current tissue clearing methods can be categorized into three groups: hydrophobic^24^, hydrophilic^25^, and hydrogel-based methods^26,27^, CUBIC^28^, DISCO^29^ and MAP^30^; they are the three the most representative methods. Among them, hydrophobic methods have better tissue clearing ability, hydrophilic methods are better at preserving tissue structures and endogenous fluorescent proteins, and hydrogel-based methods are capable of expanding the tissue structures isotopically, enabling the resolving of finer structural details with the same optical imaging method^31^. In our previous research, we reported an enhanced CUBIC (eCUBIC) method for tissue clearing and a Clearing and Magnification Analysis of Proteome (CMAP) method for tissue expansion^32^. We showed that the combination of eCUBIC, CMAP, and tiling light sheet microscopy techniques enable us to decode large neural networks with spatial resolution from micron to sub-100 nm level by studying the mouse spinal cord locomotive neural network.

Therefore, in this study, we studied five less well-known descending tracts originating from the cerebellum, midbrain and, hindbrain in mouse respectively by sparsely labelling the corresponding descending neurons through virus injection and imaging the cleared and expanded mouse brains and spinal cords using tiling light sheet microscopy. We obtained the 3D projection patterns of these tracts by imaging the cleared spinal cords and we traced dozens of axons in each tract to understand the branches’ characteristics of the tracts. We further investigated the existence of monosynaptic connections between descending tracts and the spinal cord motor neurons by imaging the expanded spinal cords. Our results not only provide novel 3D morphological information for the understanding of descending tracts in mouse, but also provide an accessible resource to understand the descending tracts in depth and contribute new methods to the study of the descending tracts.

## Results

### Distribution of the descending neurons in the mouse brain

**Figure 1:**
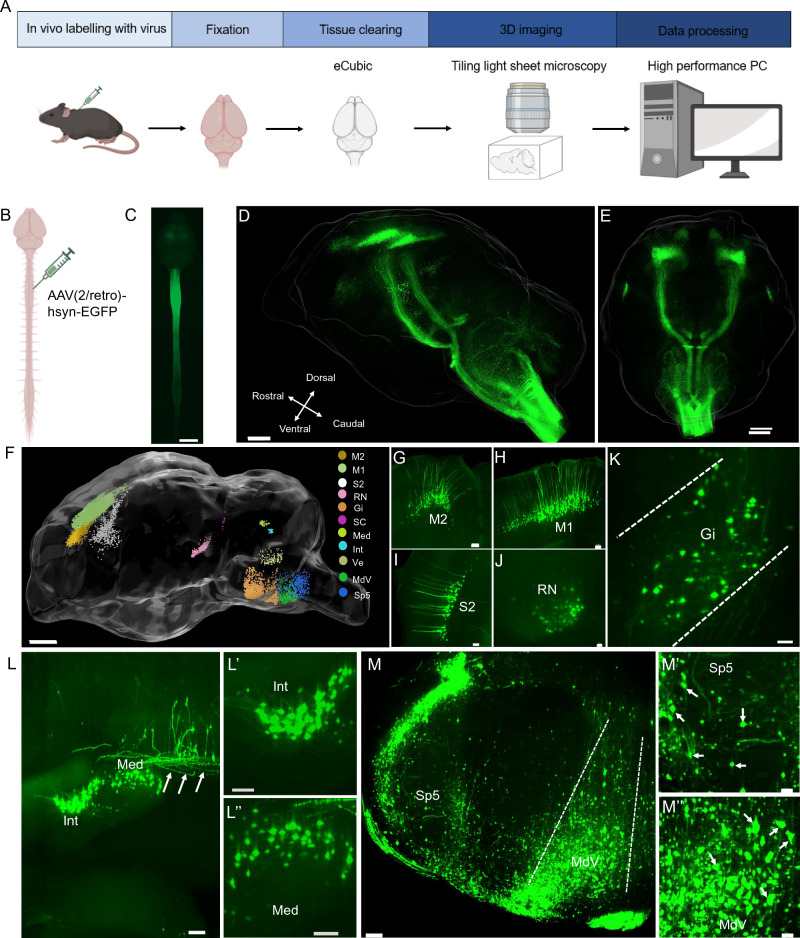
Retrograde labelling of the brain regions project to the mouse spinal cord. (**A**) Pipeline for detection of descending tracts project from the brain to the spinal cord. Retrograde virus is delivered to the spinal cord of mice, followed 3–4 weeks later by brain clearing, tiling light sheet microscopy imaging and data analysis. The schematic diagram was created using BioRender software (https://www.biorender.com). (**B**) Specific viral labelling strategy to deliver AAV(2/retro)-hSyn-EGFP into the cervical spinal cord. (**C**) Representative image shows specific expression of EGFP in the cervical spinal cord and brain under fluorescence stereo zoom microscopy (Zeiss Axio Zoom, V16). Scale bar: 5000 μm. (**D**, **E**) Lateral view and top view of the brain imaged under tilting light sheet microscopy after tissue clearing. Scale bar: 1000 μm. (**F**) Representative image shows the descending neurons in the brain after cell segmentation using Imaris. To study the specific nuclei projecting to the spinal cord, we analysed the unilateral brain regions using Imaris software carefully according to the Allen brain map. Several cell regions are labelled using AAV(2/retro)-hSyn-EGFP injecting into the cervical spinal cord. M1, primary motor cortex; M2, secondary motor cortex; S2, secondary somatosensory cortex; RN, red nucleus; Gi, gigantocellular reticular nucleus; Ve, vestibular nucleus; SC, superior colliculus; Med, medial cerebellar nucleus; Int, interposed cerebellar nucleus, anterior part/posterior part. MdV, medullary reticular nucleus, ventral part; Sp5, Spinal trigeminal nucleus. Scale bar: 1000 μm (**G**-**K**) Coronal views of dominant brain regions project to the spinal cord: motor cortex (**G**-**H**), red nucleus (**J**) and GI (**K**). Scale bar: 200 μm. (**L**) Coronal views of the labelled region in the cerebellum, interposed cerebellar nucleus (L, L’) and medial cerebellar nucleus (L, L’’). White arrows in (**L**) show both nucleus trajectories extending to the contralateral cerebellum via the fasciculus descending to the spinal cord. Scale bar: 500 μm. (**M**) Coronal views of the labelled region in the hindbrain, spinal trigeminal nucleus (M, M’) and MdV (M, M’’). Scale bar: 200 μm.

Movement commands are delivered from the brain to the spinal cord by descending tracts originating from various brain regions. To verify the 3D distribution of the descending neurons in the mouse brain, we first injected AAV2-retro-EGFP bilaterally into the mouse cervical spinal cord to label the descending neurons. The mouse brain was dissected, cleared, and imaged in 3D four weeks after the injection (Fig. 1A,C). Consistent with previous studies^17,33^, retro-labelled neurons were observed within the cortex, red nucleus, vestibular nucleus, and reticular formation by analysing four brains (Fig. 1D,K, Supplementary video (1), thus affirming the reliability of this viral tool for labelling descending neurons. Apart from those well-known descending tracts, we also observed retro-labelled neurons within deep cerebellum nuclei, namely the medial and interposed cerebellar nucleus (Fig. 1F,L). Through fasciculus, the medial and interposed cerebellar nucleus neural trajectory extended to the contralateral cerebellum and then descended to the spinal cord (Fig. 1L). Additionally, the neurons in spinal trigeminal nucleus (Sp5, Fig. 1F,M) and medullary ventral reticular formation (MdV, Fig. 1F,M) were also observed. Additionally, we counted the labelled neurons of these origins of unilateral brain (Supplementary Fig S1).

In the previous study, we have studied the projection patterns of three dominant descending tracts, containing the cortex, red nucleus and reticular formation^34^. In this study, we focused on these less-studied descending pathways to analyse the projection patterns and axon features in the spinal cord using high -magnification 3D imaging microscopy and imaging.

### Axonal projections of the medial cerebellar nucleus neurons in the spinal cord

**Figure 2:**
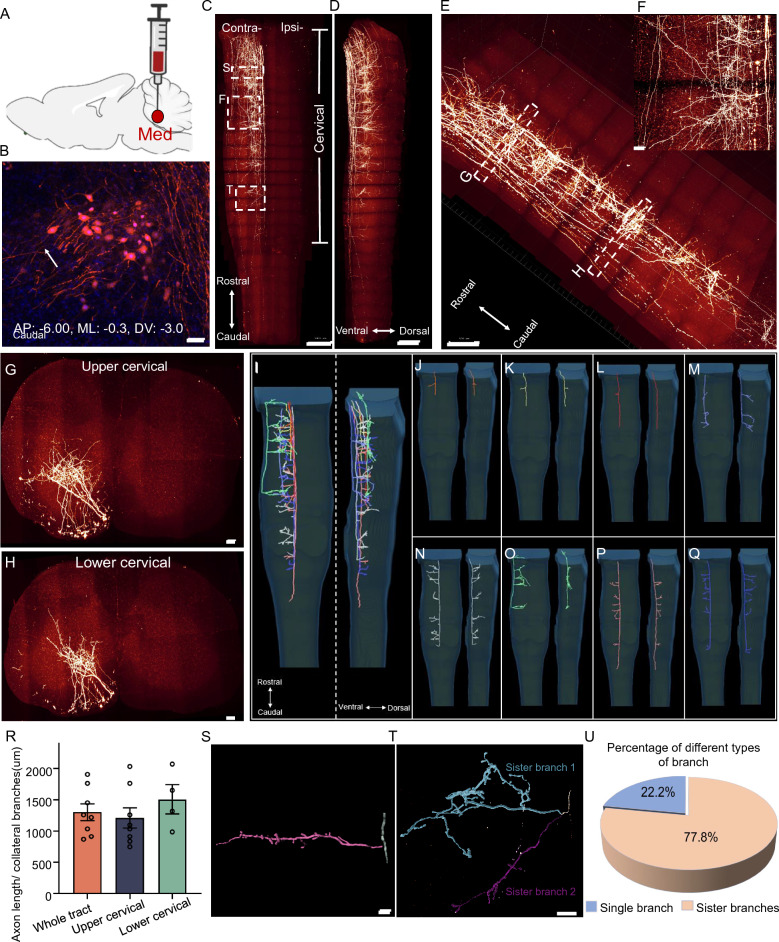
The morphology of medial cerebellar neuronal axons in the mouse spinal cord. (**A**) Schematic diagram to show the injection site, medial cerebellar nucleus (injection area: AP: − 6.00, ML: − 0.3, DV: − 3.0). The schematic diagram was created using BioRender software (https://www.biorender.com). (**B**) Representative image of the injection site. Scale bar: 50 μm. (**C**, **D**) Horizontal and lateral 3D views of the Med axonal projection pattern in the spinal cord. Scale bar: 1000 μm. (**E**) Magnified view of the cervical spinal cord. Scale bar: 500 μm. (**F**) Magnified image of axon branch details. Scale bar: 100 μm. (**G**, **H**) Coronal view of the upper and lower cervical spinal cord. Scale bar: 100 μm. (**I**–**Q**) The horizontal (left panel) and lateral (right panel) view of axons traced by Amira software in the spinal cord. (**I**) Merged image shows all the traced axons in one spinal cord. (**J**–**Q**) Morphology of individually traced axons in the spinal cord. (**R**) Analysis of collateral branch density in different areas. (**S**, **T**) Representative image of a single collateral branch and “sister” branchs. Scale bar: 100 μm. (**U**) Percentage of single collateral branches and “sister” branches.

The cerebellospinal tracts, including the axonal projections from the medial and interposed cerebellar neurons, play essential roles in forelimb movement and skilled locomotor learning^17,35,36^. We first studied the projection pattern of medial cerebellar nucleus neurons in the mouse spinal cord. The neurons were sparsely labelled by injecting a viral mixture solution of AAV2/9-hSyn-cre and AAV-hSyn-DIO-tomato at a ratio of 1:2. Our results showed that most axons of medial cerebellar nucleus neurons descend along the middle ventral side of the spinal cord and terminate at the contralateral side of the cervical and upper thoracic region of the spinal cord (Fig. 2A–D, Supplementary video (2). The majority of axons extended along the middle ventral, with only few axons projected along the lateral ventral region of spinal cord (Fig. 2E,F). The collateral branches of axons extended to the edge of the ventral region of both upper cervical and lower cervical segments (Fig. 2G,H).

Furthermore, we traced the axons from the trunk to the furthest branch terminal manually (Fig. 2I, Supplementary video (3) and found two types of projections among the traced individual axons. One type of axons only projects to upper cervical region (Fig. 2J–M) and the other projects to both upper and lower cervical regions (Fig. 2N–Q). We also analysed the density of collateral branches in upper and lower cervical regions and found that the density of collateral branches was higher in the upper cervical region than that in the lower cervical region (Fig. 2R). Interestingly, we observed two types of collateral branches in the traced axons, single collateral branch (Fig. 2S) and “sister” collateral branch (Fig. 2T). The “sister” collateral branch was those axons that divided into two branches immediately after the branch node; these have not been reported before. Both “sister” branches could be extended far away, which was obviously different from the single collateral branch type. We further found more frequent appearance of “sister” collateral branches than single collateral branches along the tract. We counted the number of “sister” branches on all the traced axons and found that ~ 77% collateral branches were “sister” branches (Fig. 2U). Moreover, we also noticed that the collateral branches on the same axon are always the same type, which means that either all collateral branches are all single branch or all are “sister” branches on the same axon (Fig. 2M–Q).

### Axonal projection pattern of interposed cerebellar nucleus neurons in the spinal cord

**Figure 3:**
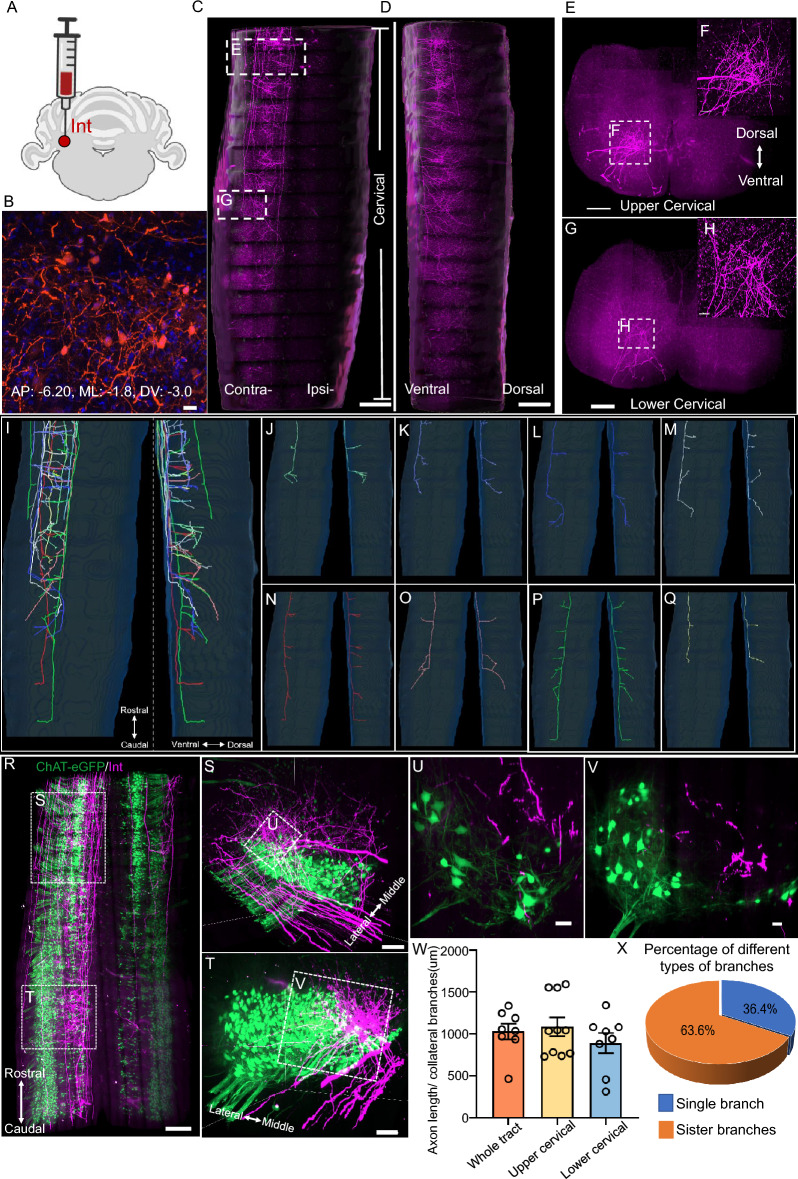
The morphology of interposed cerebellar neuronal axons in the mouse spinal cord. (**A**) Schematic diagram to show injection site, interposed cerebellar nucleus (injection area: AP: − 6.20, ML: − 1.8, DV: − 3.0). The schematic diagram was created using BioRender software (https://www.biorender.com). (**B**) Representative image of the injection site. Scale bar: 20 μm. (**C**, **D**) The horizontal (**C**) and lateral (**D**) view of interposed cerebellar axonal projection pattern in the spinal cord under a 3D view. Scale bar: 1000 μm. (**E**, **G**) Coronal view of the axons morphology in the upper (**E**) and lower (**G**) cervical spinal cord with 300 μm MIP. Scale bar: 100 μm. (**F**, **H**) Magnified images show the details of axon branches in E and G, respectively. Scale bar: 100 μm. (**I**–**Q**) Traces of individual axons in the spinal cord. (**R**) The horizontal view of the expanded cervical region of the spinal cord. A mixture of AAV2-cre/AAV2-DIO-tdTomato was injected into the interposed cerebellar area of ChAT-eGFP mice, followed by perfusion, tissue expansion with CMAP and tiling light sheet microscopy imaging. (**S**, **T**) Magnified images show the lateral view of different regions in the cervical spinal cord in (**R**). Scale bar: 100 μm. (**U**, **V**) Coronal view of ~ 3 μm MIP images in (**S**) and (**T**). Scale bar: 50 μm. (**W**) Analysis of collateral branch density in different areas. (**X**) Percentage of single collateral branches and “sister” branches.

Next, we studied the projection of interposed cerebellar nucleus neurons (Fig. 3A,B). Most axons originating from the interposed cerebellar nucleus crossed to the contralateral side in the cerebellum, descended along the ventral side of the spinal cord, and terminated in both the upper and lower cervical regions (Fig. 3C,D). The axon terminated typically at the laminae VII and VIII regions of the spinal cord (Fig. 3E–H). By tracing axons individually, we found that the collateral branches were evenly distributed in the upper cervical and lower cervical segments (Fig. 3I–Q,W). We also observed abundant “sister” collateral branches, which made up approximately 63% of all collateral branches in all traced axons (Fig. 3X).

Next, we explored whether the axons had monosynaptic connections with the spinal cord motor neurons. We injected mixture solutions of AAV2/9-hSyn-cre and AAV-hSyn-DIO-tomato into the interposed cerebellar nucleus of ChAT-eGFP mice and imaged the expanded spinal cords ~ 6 weeks after the injection using tiling light sheet microscopy (Fig. 3R). We did not find any monosynaptic connections in either upper or lower cervical regions between the axon terminals and the motor neurons (Fig. 3S–V), which hinted that neurons in Interposed cerebellar nucleus could not directly drive motor neurons.

### Axonal projections of the spinal trigeminal neurons in the spinal cord

**Figure 4:**
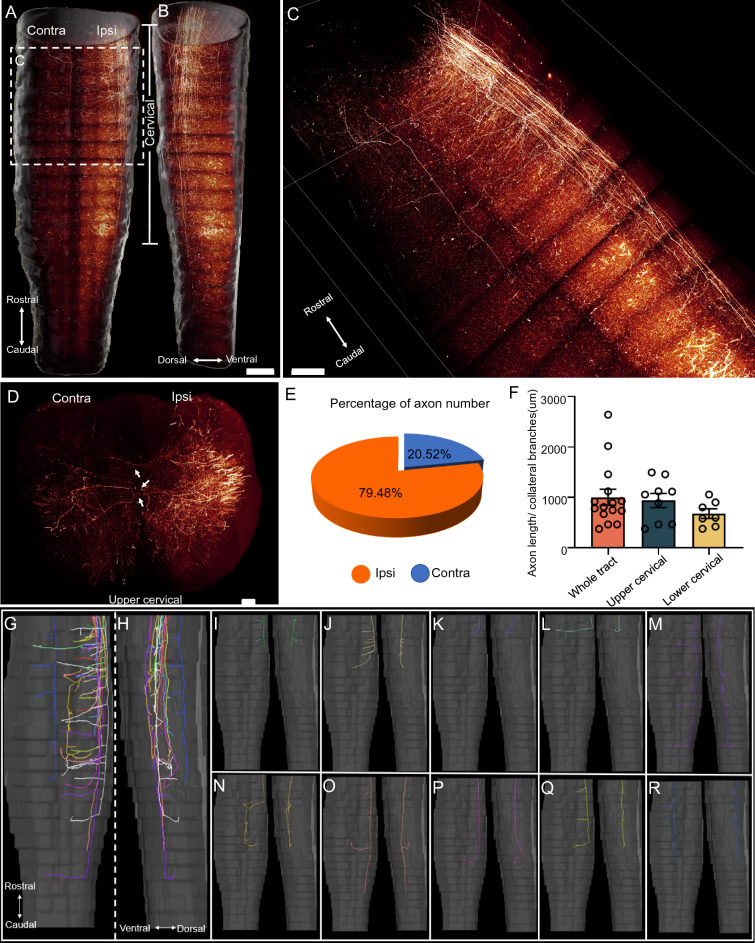
The morphology of spinal trigeminal nucleus neuron axons in the mouse spinal cord. (**A**, **B**) Horizontal (**A**) and lateral (**B**) views of the spinal trigeminal nucleus axonal projection pattern in the spinal cord by 3D volume rendering. Scale bar: 1000 μm. (**C**) Magnified view of the area indicates in (**A**). Scale bar: 1000 μm. (**D**) Coronal view of the cervical spinal cord. Scale bar: 200 μm. (**E**) Percentage of branches in the contralateral and ipsilateral spinal cord. (**F**) Percentage of collateral branches in different areas of the spinal cord. (**G**–**R**) Horizontal and lateral views of axons traced in the spinal cord using Amira.

The spinal trigeminal nucleus is a small motor centre in the hindbrain. A prior study has reported that labelled neurons in the spinal trigeminal nucleus can be detected by injecting the rabies into the mouse biceps^37^. However, they did not study the pattern of the spinal trigeminal axons in the spinal cord. In this study, we investigated the projections of the spinal trigeminal neurons in mouse by injecting anterograde virus into the spinal trigeminal nucleus. The results showed that the axons projected along the dorsolateral side of the spinal cord and terminated in the cervical or upper thoracic regions (Fig. 4A–C). Nearly 80% of the collateral branches projected to the ipsilateral region and 20% project to the contralateral region in all labelled axons (Fig. 4D,E). We found that the branches were distributed in the dorsolateral cervical segment (Fig. 4D). Unexpectedly, some branches crossed the midline and terminated at the contralateral spinal cord (Fig. 4D). The observation was further confirmed by the tracing results of individual axons manually (Fig. 4G–R). Two different types of axonal projections were observed, that were terminated in the upper cervical (C1-C4, Fig. 4I–L) and lower cervical regions (C5-C8, Fig. 4M–R) respectively. According to the traced results, we finally counted the density of axons in the upper and lower cervical segments, finding that the branch intensity was higher in the lower cervical segment, while the branches in the upper cervical segment had comparable intensity to that in all the projection areas (Fig. 4F).

Meanwhile, we traced some interesting axons which could cross the midline and get extended from the ipsilateral to contralateral side (Fig. 4L–M). In addition, we also observed an interesting axon in the lower cervical projection pattern, which originated from the lateral upper cervical segment, but turned to the midline in the lower cervical segment and then extended lots of branches (Fig. 4N).

### Axonal projections of the ventral medullary reticular formation neurons in the spinal cord

**Figure 5:**
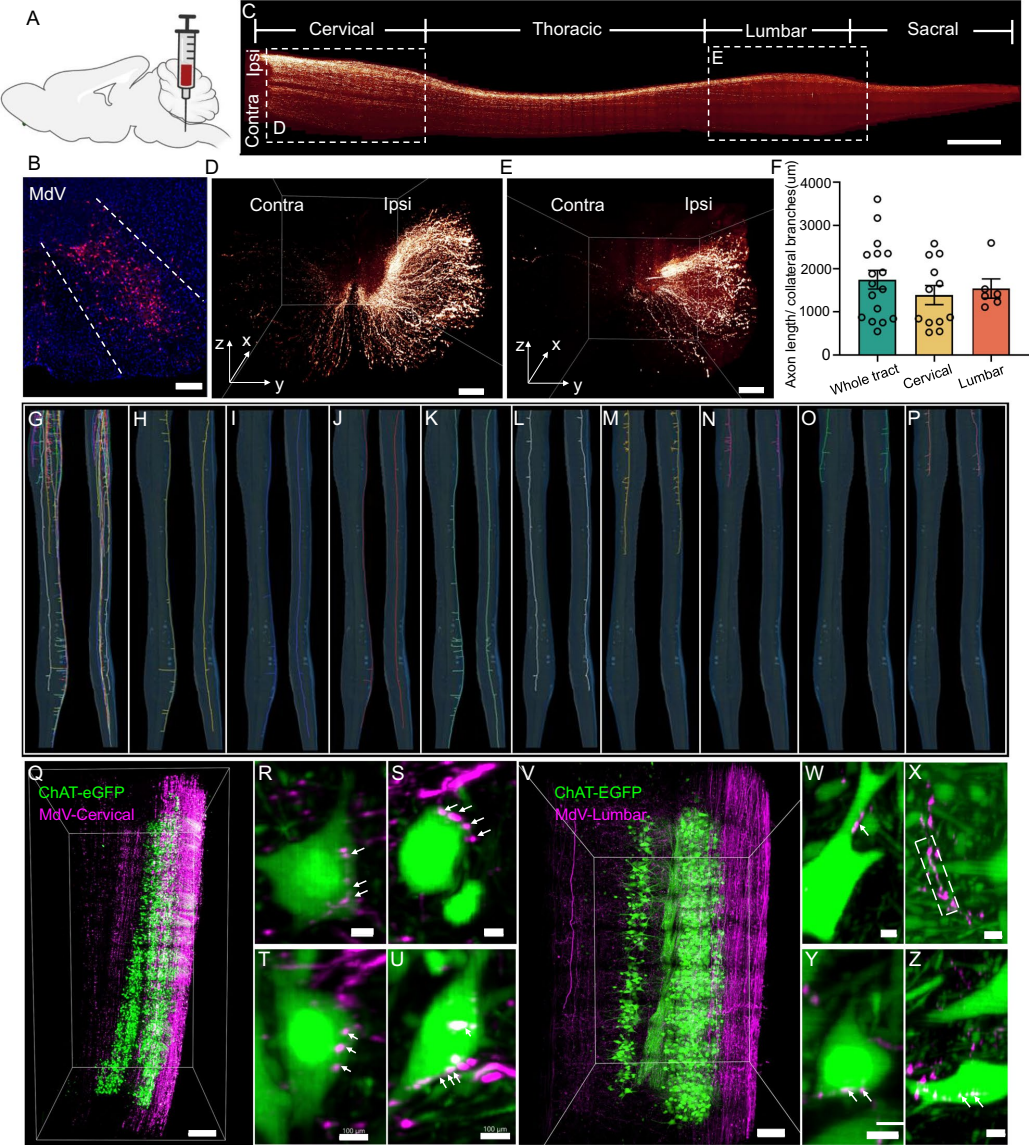
The morphology of ventral medullary reticular nucleus neuronal axons in the mouse spinal cord. (**A**) Schematic diagram of the injection site. (**B**) Representative image of the injection site. Scale bar: 500 μm. (**C**) Representative image of ventral medullary reticular formation axons in the whole spinal cord. Scale bar: 1000 μm. (**D**, **E**) 3D volume rendering shows the cervical region (**D**) and lumbar region (**E**) individually. Most axons accumulate in dorsolateral of both the cervical and lumbar regions. Scale bar: 200 μm. (**F**) Analysis of collateral branch density in different areas. (**G**–**P**) Horizontal and lateral views of axons traced in the spinal cord. (**Q**–**U**) Synaptic connections between ventral medullary reticular formation axons and motor neurons in the cervical region. (**Q**) shows the horizontal 3D volume rendering view of the expanded ipsilateral cervical of the spinal cord. Scale bar: 500 μm. (**R**–**U**) Representative images of synaptic connections between axons and motor neurons in the cervical region (scale bar: 100 μm). (**V**–**Z**) Representative images of synaptic connections in the lumbar region (scale bar: 50 μm). (**V**) shows the horizontal 3D volume rendering view of the expanded ipsilateral lumbar region of the spinal cord. Arrows and squares in (**W**–**Z**) show boutons of the axons contacting the somas or dendrites of motor neurons.

The medullary reticular formation, ventral part (MdV) is a hindbrain region that containing descending neurons drove the spinal motor neurons which innervated forelimb^37^. It was shown that glutamatergic premotor neurons in ventral medullary reticular formation received inputs from upper motor centres and were recruited for motor tasks^37^. However, there have been limited resources describing the axonal projections of ventral medullary reticular formation neurons in the spinal cord. Therefore, we studied the axonal projections of descending ventral medullary reticular formation neurons to obtain a more detailed understanding on the tract. We injected mixture solutions of AAV-Cre and AAV-DIO-tdTomato virus into the ventral medullary reticular formation of multiple mice (Fig. 5A,B) and studied the descending pattern in the whole spinal cord by imaging the cleared mouse spinal cords (Fig. 5C) and tracing dozens of individual axons.

Our results showed that the ventral medullary reticular formation axons mainly extended along the dorsolateral and ventral sides of the spinal cord (Fig. 5C–E). After the axons were traced individually, several different projection patterns were detected (Fig. 5G). In general, according to the distance of axon projection on the spinal cord, there are three projection patterns: terminated at the cervical (Fig. 5N–P), upper thoracic (Fig. 5M) and lumbar and sacral (Fig. 5G–L). The properties of axon branch properties in different projection patterns were observed and we found something interesting. For example, for those axons that terminated only in cervical or upper thoracic regions, axon branches accumulated in the cervical region (Fig. 5M–P). While, for the axons that terminated in lumbar and sacral regions, the distribution of axon branches was slightly more complicated. For instance, some axon branches were only distributed in the lumbar region (Fig. 5J), some were distributed in both thoracic and lumbar regions (Fig. 5I,K) and some were distributed in both cervical and lumbar regions (Fig. 5H,L). Our results show that the branch density is higher in the cervical region than that in the lumbar region (Fig. 5F).

Furthermore, we investigated whether ventral medullary reticular formation descending neurons drove spinal cord motor neurons directly. We injected mixture solutions of AAV-cre and AAV-DIO-tdTomato viruses into the ventral medullary reticular formation of multiple ChAT-eGFP mice. The spinal cords of the injected mice were dissected, expanded using CMAP, and imaged using tiling light sheet microscopy ~ 6 weeks after the injection. The lower cervical and lumbar regions were expanded individually (Fig. 5Q,U). Our results showed abundant monosynaptic connections between the ventral medullary reticular formation axons and the spinal cord motor neurons in both the cervical and lumbar regions, suggested that the ventral medullary reticular formation axons drove the spinal cord motor neurons directly (Fig. 5Q–Z, Supplementary video 4).

### Axonal projections of the superior colliculus neurons in the spinal cord (Tectospinal tract)

**Figure 6:**
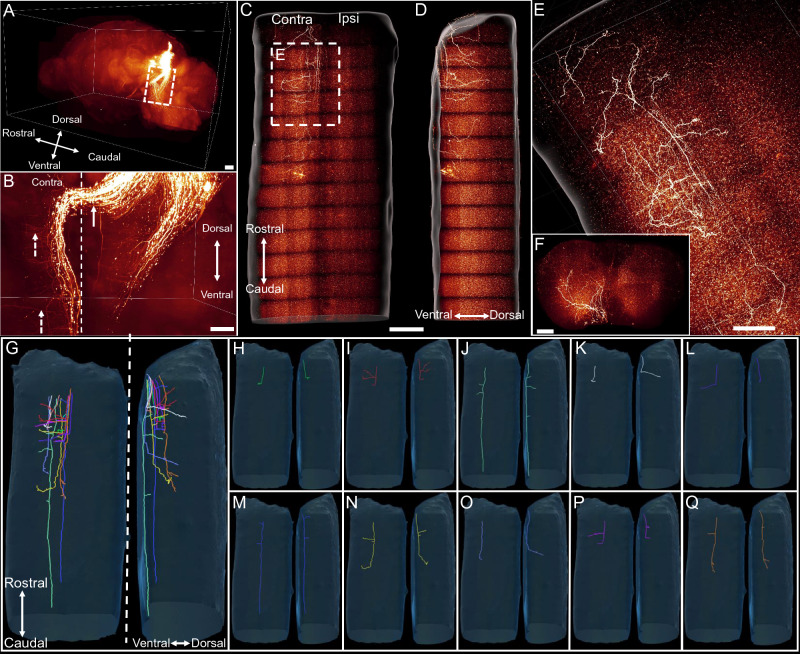
The morphology of superior colliculus neuron axons in the mouse spinal cord. (**A**, **B**) Representative images show the tract projection pattern in the brain. (**B**) Magnified image of the dashed box in (**A**). The white solid arrow shows axons crossing to the contralateral side of the brain. The dashed arrows show abundant branches of axons in the hindbrain. Scale bar: 1000 μm. (**C**, **D**) Horizontal (**C**) and lateral (**D**) views of axonal projection pattern in the spinal cord. Scale bar: 1000 μm. (**E**) Magnified view of the area indicated in (**C**). Scale bar: 1000 μm. (**F**) Coronal view of the cervical spinal cord. Scale bar: 200 μm. (**G**–**Q**) Horizontal (left panel) and lateral (right panel) views of traced axons in the spinal cord.

The tectospinal tract arises from the superior colliculus and projects to the spinal cord^18,19,38^. It is primarily involved in the head and neck movement control. In our studies, we detected rare neurons in the superior colliculus through retrograde labelling by virus injection into the spinal cord (Fig. 1E, Supplementary Fig S1). The results suggested that the superior colliculus neurons barely projected to the spinal cord in mouse, which was consistent with previous findings^8^. We further confirmed the observation by imaging the cleared spinal cords of mice labelling the SC neurons through virus injection. Indeed, most tectospinal tract axons crossed the midline in the dorsal tegmental decussation and project along the ventral side of the hindbrain with very few axons reaching the spinal cord (Fig. 6A–D). The tectospinal tract axons projected to the spinal cord descend along the ventral side of the spinal cord and terminated in the upper cervical region. The axon terminals extended to the contralateral side of the spinal cord and reach as far as the dorsal horn of the spinal cord (Fig. 6E,F). The results also showed that the tectospinal tract axons developed very few collateral branches. We traced multiple axons and counted the number of branches on the traced axons. There were less than three branches on all traced axons (Fig. 6G–Q), which was significantly less than that of the axons of other tracts. The result also was in sharp contrast with the large number of branches in the hindbrain (Fig. 6B). This phenotype may be related to the function of the superior colliculus, which mainly participates in the movement of the head and neck. Additionally, the branches of the axons extended to the middle and dorsal areas of the spinal cord, so that they were unlikely to connect with the motor neurons in the ventral horn of the spinal cord directly.

### Spatial distribution of ventral medullary reticular formation neurons in the brain

**Figure 7:**
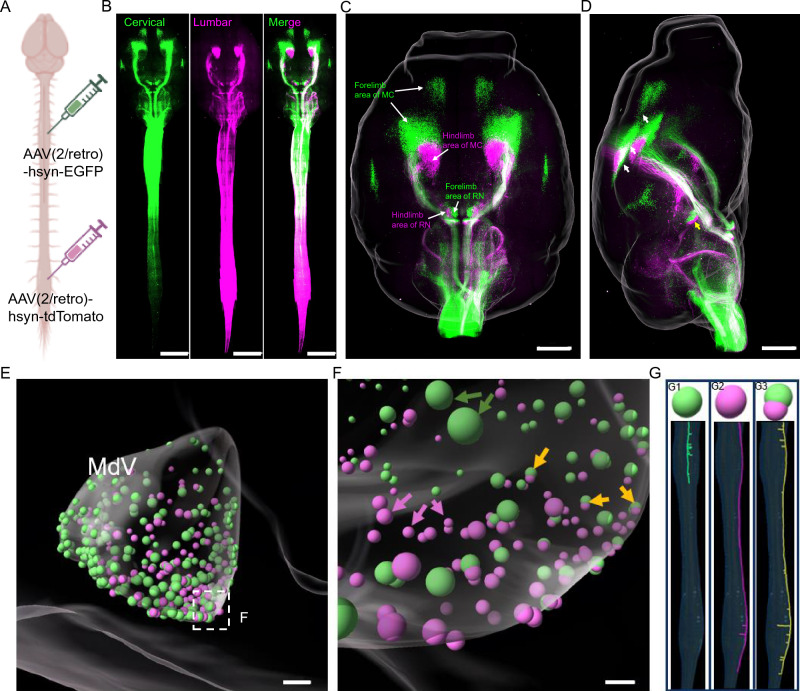
The spatial distribution of descending neurons in the brain. (**A**) Specific strategy to label the cervical and lumbar regions of the spinal cord by injecting AAV(2/retro)-hSyn-EGFP and AAV(2/retro)-hSyn-tdTomato, respectively. Scale bar: 5000 μm. (**B**) Top view of the whole CNS imaged by tiling light sheet microscopy after tissue clearing. Scale bar: 5000 μm. (**C**, **D**) Top view (**C**) and lateral view (**D**) show the distribution of different descending neurons in the brain. The motor cortex and red nucleus have distinct distributions in both the forelimb and hindlimb areas. The white arrows in (**D**) indicate the distinct distribution of the motor cortex, while the yellow arrows showed the red nucleus. Scale bar: 1000 μm. (**E**, **F**) Colocalization analysis of the descending neurons in the ventral medullary reticular nucleus by using the Imaris spot function. The green arrows indicate axons with branches only in the cervical region; the purple arrows indicate axons with branches only in the lumbar region; and the yellow arrows indicate axons with branches in both the cervical and lumbar regions. Scale bar: 100 μm. (**G**) Representative model of three types of traced axons and the corresponding soma (arrows in (**F**)) in the brain.

When the axons of ventral medullary reticular formation neurons were traced, it was observed that the labelled axons projected to the cervical or lumbar region, which made us to be interested in how those neurons with different projection patterns are distributed within the brain. In order to inspect the somatotopic arrangement of the descending neurons, we injected AAV2-retro-EGFP and AAV2-retro-tdTomato virus solutions into the cervical and lumbar regions of the same mouse spinal cord respectively (Fig. 7A) and imaged the whole cleared central nervous system (CNS) of the injected mouse using tiling light sheet microscopy (Fig. 7B). We observed spatial corresponding relationship between the descending neurons in the motor cortex and red nucleus of the brain and the cervical and lumbar segments of the spinal cord (Fig. 7C,D, Supplementary video (5), which is consistent with prior studies^9,17,33^. Next, we focused on the spatial distribution of ventral medullary reticular formation neurons and found that there was no obvious spatial distribution like that in the motor cortex or red nucleus (Fig. 7E). However, we observed that many neurons in the ventral medullary reticular formation were either GFP- or tdTomato-positive or GFP and tdTomato double -positive (Fig. 7F), which inversely demonstrates our findings that ventral medullary reticular formation neurons have complex branching patterns in different segments of the spinal cord. This further implied that our tracing results were reliable (Fig. 5G–P, Fig. 7G).

## Discussion

Descending pathways carry motor commands to the spinal cord, linking the motor plan to the task execution. In the past few decades, researchers have used retrograde virus and dye methods to label the descending tracts and study their morphology and distribution in the spinal cord via tissue sectioning and 2D imaging of discrete tissue slices. However, this method was not only laborious but only acquired incomplete projection information. Clearly, it is impossible to obtain the morphology of descending axons in detail, which is required to understand the function of the descending tract. In the present study, we demonstrated a new method to study the descending tracts comprehensively through the combination of virus labelling, tissue clearing and light sheet microscopy. First, we injected retrograde virus into the spinal cord to detect the nuclei projecting to the spinal cord directly by means of intact brain imaging. Second, we focused on five pathways and further analysed the distributions of these pathways and corresponding axon morphologies in the spinal cord under high magnification. Finally, we analysed whether the tracts had monosynaptic connections with motor neurons in the ventral horn of the spinal cord by using CMAP.

Classic anatomical studies have shown that the majority of cerebellospinal tract axons, originating from the medial and interposed cerebellar nucleus, project to the contralateral spinal cord and the axon terminals distributed in laminae VII and VIII of the cervical regions^5,17,39^. Our data showed that both the medial and interposed cerebellar nucleus pathways projected to the contralateral cervical segment of the spinal cord along the mid-ventral surface that are consistent with previous studies. The tracing results also showed for the first time that the neurons in medial and interposed cerebellar nucleus had a high ratio of “sister” branches, which was not reported before. As a medium for information transfer, axons are responsible for transmitting information from the soma to the axon terminals and further to the next level of neurons. Therefore, we speculated that the “sister” branches may enlarge the dominant area, which is conductive to information transfer. The medial cerebellar nucleus axon branches were more densely distributed in the upper cervical region, while the interposed cerebellar nucleus axon branches were more densely distributed in the lower cervical region, which may indicate different functions and abilities of the two pathways. However, the reason for the difference requires further functional analysis between of the both pathways. Next, we have imaged the intact cervical region of the spinal cord after injecting the anterograde virus into ChAT-eGFP mice. No direct synaptic connection was detected between the descending axons and the motor neurons neither in upper cervical nor lower cervical segment, which was consistent with the previous study^17^.

In our study, we found that the majority of spinal trigeminal axons terminated at the cervical region and that only few axons were extended to the thoracic spinal cord in mice, which was consistent with the findings in the human brain^21^. Previous study has shown that spinal trigeminal neurons can be labelled by injecting the monosynaptic rabies virus into the forelimb muscle, which indicated that the spinal trigeminal is associated with forelimb control. This might explain why most labelled axons in our study terminated in the lower cervical segment and had a high density of axon branches. In this study, we also observed several axon branches in the upper cervical region, which may hint at the role of spinal trigeminal in face and head control. Surprisingly, we first traced several spinal trigeminal axons that crossed the midline to the contralateral side in the spinal cord, which reflected the complexity and diversity of the spinal trigeminal axon morphology and distribution, although the function of the different types of axons is not yet clear.

In 2014, Maria et al. reported that the ventral medullary reticular formation descending neurons were important for skilled forelimb motor behaviours^37^. In this study, we imaged the whole spinal cord, and observed that the ventral medullary reticular formation axons mainly projected to the cervical and lumbar regions, which may suggest its function in forelimb and hindlimb motor control. Meanwhile, the higher collateral branch density in the cervical region may suggest a different degree of participations of the ventral medullary reticular formation neurons in the forelimb and hindlimb motor control. In Esposito et al.’s study, they only reported synaptic connection between the ventral medullary reticular formation axons and the motor neurons in the cervical region^37^; however, in this study, we found that the ventral medullary reticular formation axons had monosynaptic connections with the spinal cord motor neurons in both the cervical and lumbar regions.

Finally, we visualized the relative spatial distribution of neurons in the brain that project to the cervical and lumbar spinal cord combined with AAV2-retro virus from a 3D view. Consistent with previous studies, the descending neurons from the motor cortex and red nucleus had an apparent spatial distribution which controlled the forelimbs and hindlimbs^17,33^, while, there was no obvious spatial distribution of ventral medullary reticular formation neurons. Surprisingly, we found that several neurons were both GFP- positive and tdTomato- positive in the ventral part of the ventral medullary reticular formation, which demonstrated our findings that ventral medullary reticular formation neurons had complex branching patterns in different segments of the spinal cord and these results further confirmed that our tracing results were reliable.

In summary, we first reported the full- view of the projection patterns and axon morphologies of five descending tracts in the mouse spinal cord under a 3D view. Our results both validated the reported data and uncovered new features that were not previously reported and can unlikely be observed using conventional methods. More importantly, we show that the 3D imaging method could be used to obtain much more detailed knowledge of projection patterns and the connectome of neurons and give insight into possible functional significance of the identified morphological connections.

## Methods and materials

### Animals and viruses

All experiments were carried out in accordance with the ARRIVE guidelines (https://arriveguidelines.org). The study was approved by the Institutional Animal Care and Use Committee (IACUC) of the Westlake University, Hangzhou, China. All methods were performed in accordance with relevant guidelines and regulations. All mice were housed on a 12 h/12 h light/dark cycle with standard chow. The ChAT-eGFP mouse line was maintained on a C57BL/6 J background and was kindly provided by Dr Liang Wang from the Zhejiang University. In this study, 20 adult male mice were used. Four mice were used to detect descending neurons by injecting retrograde virus in the cervical region of the spinal cord. Three mice were used to study the relative spatial distribution of descending neurons by injecting retrograde virus in the cervical and lumbar regions, respectively. From two to four mice were used to analyse each tract (Med: 2; Int: 3, Sp5: 2; MdV: 4; superior colliculus: 2). All AAV viruses were purchased from the Brain VTA (Brain VTA Co., Ltd., Wuhan, China). Viral particles were injected at a titer of 1-5E12 genome copies per mL.

### Surgery and virus injection

Spinal cord injection: the mouse was anaesthetized with intraperitoneal injection of amobarbital sodium. The injection was performed according to a previously described method^40^. Briefly, a small incision was made in the skin. The muscle and adipose tissues underneath were teased to expose the vertebral column. The tissue joining the dorsal processes of consecutive vertebrae was removed. The vertebral surfaces were cleaned with a fine tweezer and the vertebrae were gently separated to expose the dorsal surface of the spinal cord for injection. A stereotaxic apparatus (RWD Technology Corp., Ltd) was used for virus injection. AAV(2/retro)-hSyn-eGFP or AAV(2/retro)-hSyn-tdTomato solutions was injected through a pulled glass needle at a depth of 0.8–1 mm below the dorsal surface. The viral particles were injected at a rate of 50 nL/min with 1 µL volume per segment at three sites. The incision was sutured with absorbable sutures and closed with Vetbond after injection. The mouse received an intradermal injection of meloxicam SR for analgesia and was placed on a heating pad until woke up before returned to the home cages. Cervical region (C5-C8) and lumbar region (L2-L4) injections were performed in line with the same protocol.

Brain virus injection: a stereotaxic apparatus (RWD Technology Corp., Ltd) was used for the virus injection. The virus solution was injected through a pulled glass needle via pressure injection. Virus solutions with rAAV2-hSyn-cre and rAAV2-DIO-tdTomato mixed at a ratio of 1:1 to 1:5 was used for injection. The following craniotomies were used for the injection of the corresponding target regions: Med: AP − 6.00, ML − 0.3, DV − 3.0; Int: AP − 6.20, ML − 1.8, DV − 3.0; superior colliculus: AP − 4.50, ML − 0.5, DV − 1.2; Spinal trigeminal: AP − 6.00, ML − 1.5, DV − 4.0; MdV: AP − 7.20, ML − 0.5, DV − 3.2 from pia). The viral particles were injected at a rate of 10 nL/min with 100–200 nL volume per injection site. Following injections, the skin incision was closed using Vetbond and the mouse was returned to the home cage after injection.

### Histology and immunofluorescence

The anaesthetized mouse was perfused with PBS followed by cold 4% paraformaldehyde (PFA). The whole CNS was harvested and postfixed in cold 4% PFA overnight under gentle shaking. The coat of the spinal cord was peeled softly with tweezers after washing several times with PBS. To verify the labelling of the desired brain neurons, the mouse brain was dissected and 50 μm brain slices were made using a vibratome and incubated in DAPI (1:5000). The prepared brain slices containing the injected regions were examined under a confocal microscope (Zeiss LSM 980). The mouse spinal cord was cleared or expaned for 3D imaging if the desired brain neurons were labelled correctly.

### Tissue clearing and tissue expansion

The tissue was treated with eCUBIC and CMAP as previously reported^32^. Preparation of cleared spinal cord and brain (eCUBIC): The tissue was immersed in delipidation solution (15 wt% urea, 10% wt% N-butyldiethanolamine, 10 wt% Triton X-100 and 65 wt% ddH_2_O) at 37 °C for 3–5 days under gentle shaking until the tissue became transparent. The delipidation solution was replaced every 24 h. Following that, the delipidated tissue was transferred to and kept in RI matching buffer (25 wt% urea, 22.5 wt% sucrose, 22.5 wt% antipyrine, 10 wt% triethanolamine) at 25 °C for 2 days with daily changes. Finally, the tissue was embedded in a 2% agarose gel made with RI matching buffer. The embedded tissue was mounted on the sample holder and imaged in silicone oil under tiling light sheet microscopy.

Preparation of expanded spinal cord samples (CMAP): After clearing the sample following the procedure described above, the tissue was washed with 0.01 M PBS for 2–3 h at room temperature under gentle shaking. The spinal cord was incubated with monomer solution after washing (30% Acrylamide, 0.075% N, N-Dimethylacrylamide, 10% Sodium acrylate, 0.5% 2,2’-Azobis dihydrochloride in 0.01 M PBS) at 4 °C for 2 days under gentle shaking. Next, the spinal cord was polymerized and embedded using ultraviolet light on ice. Finally, the polymerized sample was placed in filtered DI water at room temperature for 2 days for subsequent expansion with daily DI water changes. The expanded sample was glued on an iron sheet, mounted on the sample holder, and imaged using DI water as the imaging buffer.

### Imaging and statistical analysis

The imaging experiment was performed using a previously reported tiling light sheet microscope^34,41,42^. An Olympus MVPLAPO 1X lens was used to the whole brain. Images were captured under 6.3X magnification. A Nikon Plan Apo 10X Glyc lens was used to the whole spinal cord. In addition, An Olympus XLPLN10XSVMP objective was used to image expanded samples.

3D images were aligned and merged semiautomatically using Amira software. Axon tracing was performed manually using the Amira filament tracing package. The axons were traced from the trunk to the furthest branch terminal. Cell segmentation was performed using the spot function of Imaris 9.3. The density of collateral branches was analysed using GraphPad Prism 9. All data are presented as the mean ± s.e.m. The statistical analysis was performed using unpaired Student’s t-tests.

### Supplementary Information

Supplementary Legends.

Supplementary Figure S1.

Supplementary Video 1.

Supplementary Video 2.

Supplementary Video 3.

Supplementary Video 4.

Supplementary Video 5.

## Data Availability

The data are available upon request from the corresponding author upon reasonable request.

## Abbreviations

CMAP: Clearing and magnification analysis of proteome
M1: Primary motor cortex
M2: Secondary motor cortex
S2: Secondary somatosensory cortex
RN: Red nucleus
Gi: Gigantocellular reticular nucleus
Ve: Vestibular nucleus
SC: Superior colliculus
Med: Medial cerebellar nucleus
Int: Interposed cerebellar nucleus
MdV: Medullary reticular nucleus, ventral part
Sp5: Spinal trigeminal nucleus
Contra: Contralateral
Ipsi: Ipsilateral
C1: The 1st cervical segment
C4: The 4th cervical segment
C5: The 5th cervical segment
C8: The 8th cervical segment
T1: The 1st thoracic segment
T2: The 2nd thoracic segment
