# Supplementary material for: Morphological analysis of descending tracts in mouse spinal cord using tissue clearing, tissue expansion and tiling light sheet microscopy techniques
Source: Sci Rep. 2023 Sep 30;13:16445. doi: 10.1038/s41598-023-43610-z (PMC10542777; doi:10.1038/s41598-023-43610-z)
Supplement: Supplementary file 1 — Supplementary Legends. [file 41598_2023_43610_MOESM1_ESM.docx]

Morphological Analysis of Descending Tracts in Mouse Spinal Cord Using Tissue Clearing, Tissue Expansion and Tiling Light Sheet Microscopy Techniques

Jiongfang Xie^1,2,3, #, *^, Ruili Feng^1,2,3, #^, Yanlu Chen^2,3^, Liang Gao^2,3*^

1. Fudan University, Shanghai, 200433, China.

2. Key Laboratory of Structural Biology of Zhejiang Province, School of Life Sciences, Westlake University, Hangzhou, Zhejiang, 310024, China

3. Westlake Laboratory of Life Sciences and Biomedicine, Hangzhou, 310024, China.

* The corresponding authors should be addressed Jiongfang Xie at xiejiongfang@westlake.edu.cn and Liang Gao at [gaoliang@westlake.edu.cn](mailto:gaoliang@westlake.edu.cn).

# The authors contribute equally

**Supplementary figure legends**

**Supplementary Fig 1**

The quantification of retro-labelled neural number in specific nuclei in unilateral brain. MC, motor cortex; RN, red nucleus; Gi, gigantocellular reticular nucleus; Ve, vestibular nucleus; Sp5, Spinal trigeminal nucleus; Int, interposed cerebellar nucleus, anterior part/posterior part; Med, medial cerebellar nucleus; SC, superior colliculus; MdV, medullary reticular nucleus, ventral part.

**Supplementary video legends**

**Supplementary video 1**

3D visualization of descending neurons labelled in whole brain by bilateral injection in cervical region.

**Supplementary video 2**

3D visualization of medial cerebellar nucleus axon projections and collateral branches.

**Supplementary video 3**

3D visualization of one medial cerebellar nucleus axon traced result.

**Supplementary video 4**

3D visualization of monosynaptic connections between ventral medullary reticular formation axon terminations and motor neurons.

**Supplementary video 5**

3D visualization of descending neurons that project differentially to cervical versus lumbar spinal cord.
