# Supplementary figures and images for: Morphological analysis of descending tracts in mouse spinal cord using tissue clearing, tissue expansion and tiling light sheet microscopy techniques
Source: Sci Rep. 2023 Sep 30;13:16445. doi: 10.1038/s41598-023-43610-z (PMC10542777; doi:10.1038/s41598-023-43610-z)

## Slide 1
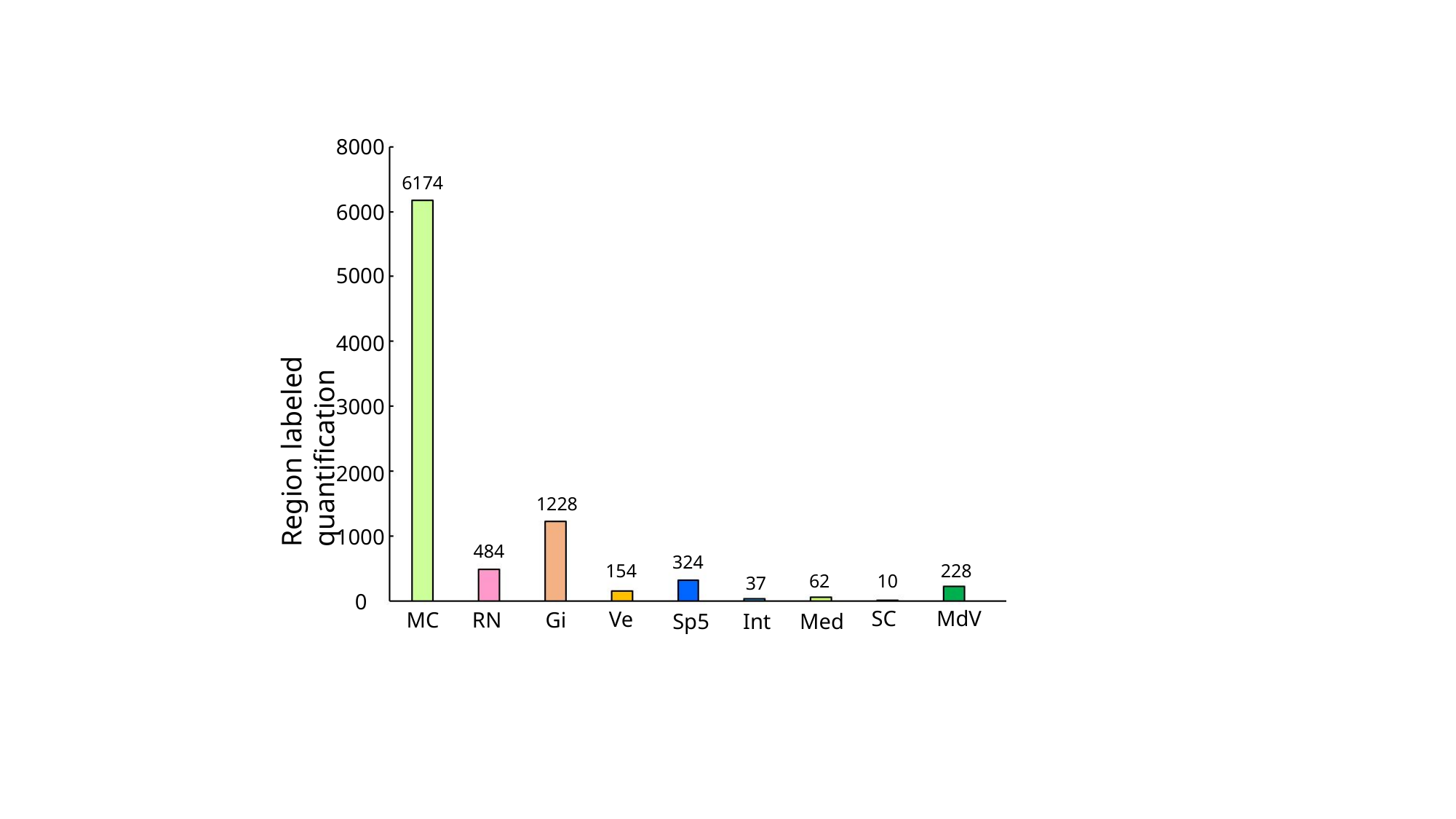

8000
6174
6000
5000
4000
3000
2000
1228
1000
484
324
228
154
10
62
37
0
SC
MdV
Ve
MC
RN
Gi
Med
Int
Sp5
Region labeled quantification

Supplement: Supplementary file 2 — Supplementary Figure S1. [file 41598_2023_43610_MOESM2_ESM.pptx]
